# Training drives turnover rates in racehorse proximal sesamoid bones

**DOI:** 10.1038/s41598-022-26027-y

**Published:** 2023-01-27

**Authors:** Sarah K. Shaffer, Susan M. Stover, David P. Fyhrie

**Affiliations:** 1 Department of Orthopaedic Surgery, School of Medicine, University of California, Davis, USA; 2 Department of Surgical and Radiological Sciences, School of Veterinary Medicine, University of California, Davis, USA; 3 Department of Biomedical Engineering, University of California, Davis, USA

**Keywords:** Medical research, Biomedical engineering, Mechanical engineering, Bone

## Abstract

Focal bone lesions are often found prior to clinically relevant stress-fractures. Lesions are characterized by low bone volume fraction, low mineral density, and high levels of microdamage and are hypothesized to develop when bone tissue cannot sufficiently respond to damaging loading. It is difficult to determine how exercise drives the formation of these lesions because bone responds to mechanical loading and repairs damage. In this study, we derive steady-state rate constants for a compartment model of bone turnover using morphometric data from fractured and non-fractured racehorse proximal sesamoid bones (PSBs) and relate rate constants to racing-speed exercise data. Fractured PSBs had a subchondral focus of bone turnover and microdamage typical of lesions that develop prior to fracture. We determined steady-state model rate constants at the lesion site and an internal region without microdamage using bone volume fraction, tissue mineral density, and microdamage area fraction measurements. The derived undamaged bone resorption rate, damage formation rate, and osteoid formation rate had significant robust regression relationships to exercise intensity (rate) variables, layup (time out of exercise), and exercise 2–10 months before death. However, the direction of these relationships varied between the damaged (lesion) and non-damaged regions, reflecting that the biological response to damaging-loading differs from the response to non-damaging loading.

## Introduction

Physiologic loading can cause damage in bone that can be repaired by bone cells. During repair osteoclasts remove damaged tissue and form porosities. Next, osteoblasts deposit unmineralized bone (osteoid) to refill the porosities. Osteoid mineralizes in a two-stage process partially controlled by osteocytes^1–3^. A rapid primary mineralization stage brings the osteoid to 45–80% of the final mineralization level reached at the end of secondary mineralization within a few days^1–4^. The slower secondary mineralization stage increases mineral density at a decreasing rate for several years and often the new tissue will be again remodeled before it reaches the maximum possible mineralization^1–4^. In summary, damage repair temporarily increases porosity and reduces tissue mineralization, thus reducing tissue stiffness at the location where the damage occurred^5,6^. These transient changes can accelerate the progression to a clinically significant stress fracture when the same level of loading (exercise) continues during the repair process, since the reduced modulus will increase strain magnitude and promote the formation of more microdamage, leading to more damage repair in a vicious cycle. Consistent with this idea, damage removal is targeted, microdamage preferentially forms near resorption bays, and computational modeling indicates resorption bays act as stress-risers^6–9^. However, the relationship between exercise and stress fractures is further complicated by bone’s response to loading.

Loading history, strain rate and magnitude, general health status, anatomic location, and other factors affect bone formation or resorption in response to loading (or lack of loading) through both bone modeling and remodeling^10^. These processes are distinguished based on the coupling of cellular activity. During modeling, bone formation and resorption are not linked. Modeling is associated with shape changes (e.g., modifying trabecular width) and may or may not be associated with changes in bone density. During remodeling, bone resorption is followed, at the same location, by formation and the amount of tissue removed is approximately equal to the amount added. However, unequal amounts of tissue formation or resorption can occur during remodeling due to disease, endocrine changes, and other factors^11^.

Focal changes in bone tissue, consistent with damage repair and response to load, are often observed prior to stress-fracture^12–16^. A bone lesion characterized by low bone volume fraction, low mineral density, microdamage, and (if location permits) an endosteal or periosteal callus are often observed in association with stress fracture^12,16–18^. Lesions are hypothesized to occur due to the interaction between damage accumulation and repair when the bone is unable to sufficiently respond to loading and nearby dense tissue and callus are considered to be a compensatory response to loading and weakness induced by the bone lesion. Exercise likely affects the development of these lesions and subsequent fractures. In racehorses, this concept is supported by observations that stress fracture risk increases with exercise intensity^12,19^. Further, similar stress fractures are often found within athlete groups; implying a link between specific exercise types and location of the stress-fracture^20^. For example, many stress fracture sites, associated bone lesion, and fracture configurations observed in racehorses are absent in horses that do not habitually train at racing-speeds^18^. Also, in racehorses, focal bone lesions and callus are often found both at the complete fracture site and at the same anatomic location in the contralateral side of the body^12,16,17^.

The interactions between exercise-induced bone changes ("Wolff's Law") and damage repair make it difficult to determine what specific exercise regimes cause (or protect against) stress fracture^10^. Racehorses are one group where this difficulty is pronounced—as stress fractures are the most common cause of fatalities associated with horse racing^12,21–23^. For example, horses with a higher-rate of training 2–12 months prior to death and those in training for longer periods without a break have an increased stress-fracture risk^19,24^. However, the 1–2 months after returning to work are also a period of high stress-fracture risk.^12^ Therefore, it would be advantageous to determine what specific aspects of a training program are protective against lesion development and resulting stress-fractures.

**Figure 1:**
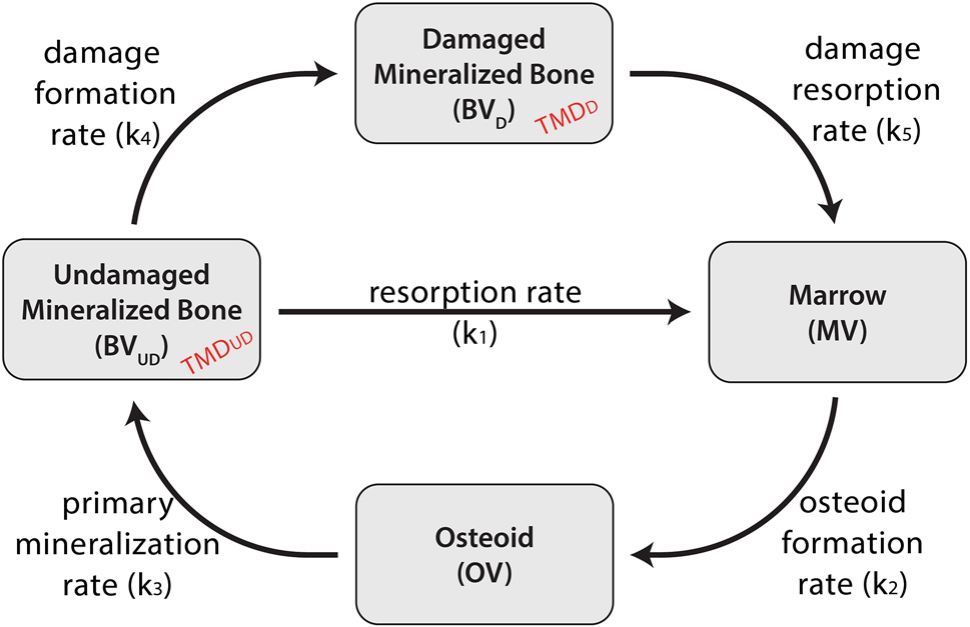
Compartment model of the bone “tissue turnover cycle”. There are four tissue volumes: damaged mineralized bone, undamaged mineralized bone, marrow, and osteoid. Each tissue type is a portion of the total tissue volume (TV) and is defined using histological features. The summation of the four volume compartments is constant, as this compartment model is closed. Tissue volumes can transform following the paths indicated by the arrows at the specified rates (*k*_*i*_). Tissue mineral density (TMD) is defined for the undamaged and damaged mineralized bone volumes.

Previously, we introduced a compartment model of bone’s “tissue turnover cycle” (Fig. 1)^25^ and used the model to organize observed relationships between racehorse proximal sesamoid bone (PSB) morphometric and training data. Exercise related proximal sesamoid bone (PSB) fracture is the most common fatal injury in many racing populations^21–23^. Consistent fracture configurations, a subchondral focus of stress remodeling and microdamage, and association with training indicate that PSB fractures are stress fractures^17,19,25–28^. We reported a focal subchondral low density and microdamaged lesion in fractured and contralateral limb intact medial PSBs of Thoroughbred racehorses; this lesion was not found in Control racehorses^17,25^. The observed differences among Case and Control PSB morphometry were site-specific and correlated with training^25^. Figure 1 shows the compartment model that relates changes in bone tissue histological measures to bone turnover rates. This model accounts for changes in bone volume fractions caused by modeling and remodeling^25^. The aims of this manuscript are to: (1) calculate steady-state rate constants for the compartment model (Fig. 1) based on previously collected histological and microcomputed tomography (μCT) data from racehorse PSBs and (2) determine the relationships between the steady-state rate constants and exercise data.

## Methods

### Study design

**Figure 2:**
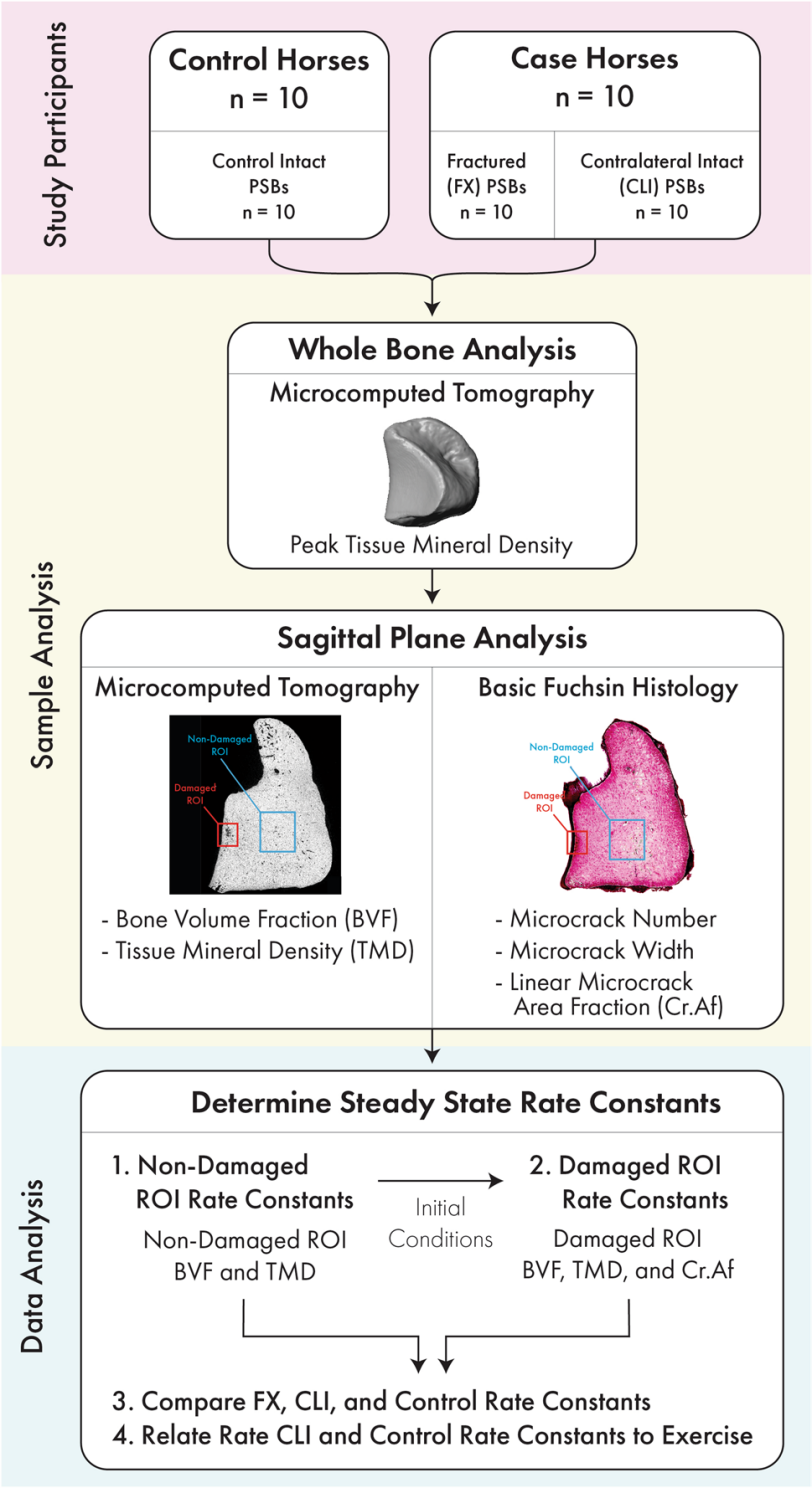
Flowchart of study methods. Study participants: Data were collected from 30 racehorse proximal sesamoid bones (PSBs). Specifically, data were measured within ten fractured medial PSBs (FX-PSB) from Case racehorses euthanized due to unilateral biaxial PSB fracture, ten contralateral limb intact medial PSBs (CLI-PSBs) from the same Case racehorses, and ten medial PSBs from Control racehorses (CTRL-PSBs) euthanized for reasons unrelated to PSB fracture^17^. All horses were in race-training at the time of death^25^. Sample analysis: The whole PSB was used to determine the peak tissue mineral density of the sample. Data was collected in two sagittal plane regions of interest (ROIs): A Non-Damaged ROI (blue) and Damaged ROI (red). These regions are described in detail in Shaffer et al., 2022^25^. Data analysis: Steady-state rate constants were first determined in the Non-Damaged ROI (1). Second, steady-state rate constants were determined within the Damaged ROI (2), using the rate constants for the Non-Damaged ROI (1) as initial conditions. Finally, the rate constants were compared among groups (3). CLI-PSB and Control PSB rate constants were related to exercise variables (4). Note that FX rate constants were excluded from this final analysis (4) because Case FX and Case CLI PSBs are from the same horses and, therefore, have the same exercise history.

The compartment model (Fig. 1) uses bone tissue types that are defined histologically or with radiographic imaging. In the following sections, we derive the relationships between morphometric data and model rate constants. Then, we calculate steady-state rate constants using previously collected morphometric data in two regions of interest (ROIs) from 30 racehorse PSBs: a Damaged and a Non-Damaged ROI (Fig. 2)^25^. The Damaged ROI either contains an identified subchondral bone lesion or was constructed at a comparable location in samples without a lesion and the Non-damaged ROI was constructed in a standardized internal trabecular region^25^. The 30 PSBs were collected at necropsy from 20 horses (10 case horses with unilateral biaxial PSB fracture, 10 control horses without PSB fracture) in three study groups (Fig. 2): case fractured (FX; n = 10), case contralateral limb intact (CLI, n = 10), and Control (CTRL, n = 10)^25^. In each ROI, the steady-state rate constants were compared among the three groups and related to exercise (Fig. 2).

### Compartment model description

This compartment model (Fig. 1) separates bone tissue into four types that fill the tissue volume (*TV*): undamaged mineralized bone (*BV*_*UD*_), damaged mineralized bone (*BV*_*D*_*)*, osteoid (*OV*), and marrow or void space (*MV*) (Eq. 1a). Tissue types transform between compartments at the given transfer rates (k_1_–k_5_; Fig. 1 and Table 1) and rates must be non-negative to prevent physiologically nonsensical negative compartment volumes^29,30^. Each compartment defines a volume within the *TV*, so, internal surfaces are not defined and volume changes due to modeling and remodeling cannot be distinguished. This is allowable because modeling and remodeling have the same volume-averaged effect within the *TV*.

**Table 1:** Model variables; all predefined values (or ranges) are indicated and variables that were solved for are indicated by “unknown”.

Parameter	Description	Value	Units
*TMDo*	Minimum tissue mineral density during secondary mineralization	540	mgHA/ccm
*TMDmax*	Maximum tissue mineral density during secondary mineralization	1163.7	mgHA/ccm
*k*_*m*_	Secondary mineralization rate constant	0.00505	Days^−1^
*k*_*1*_	Undamaged mineralized tissue resorption rate	Unknown	Days^−1^
*k*_*2*_	Osteoid formation rate	Unknown	Days^−1^
*k*_*3*_	Primary mineralization rate	1.024, 10, or 100	Days^−1^
*k*_*4*_	Damage formation rate	Unknown	Days^−1^
*k*_*5*_	Damaged mineralized tissue resorption rate	Unknown	Days^−1^
*BV*_*D*_*/TV*	Damaged mineralized bone volume per tissue volume	Derived from histology	Volume/volume
*BV*_*UD*_*/TV*	Undamaged mineralized bone volume per tissue volume	Derived from histology	Volume/volume
*MV/TV*	Marrow (or void) volume per tissue volume	Derived from histology	Volume/volume
*OV/TV*	Osteoid volume per tissue volume	Derived from histology	Volume/volume

1a $$B{V}_{UD}+B{V}_{D}+OV+MV=TV$$

The model is based on observations of bone modeling and remodeling. Undamaged bone resorption (BV_UD_ to MV) occurs at the resorption rate (k_1_) and damaged bone resorption (BV_D_ to MV) occurs at the damage resorption rate (k_5_). Bone formation is represented by osteoid formation (*MV* to *OV)* at the osteoid formation rate (k_2_) followed by primary mineralization (*OV* to BV_UD_) at the primary mineralization rate (*k*_*3*_). We assume that tissue in the mineralized bone compartments (*BV*_*UD*_ & *BV*_*D*_) has completed primary mineralization and is undergoing secondary mineralization or is fully mineralized; so, *BV*_*UD*_ & *BV*_*D*_ have a mineral density that can change with time. Damage formation (*BV*_*UD*_ to *BV*_*D*_*)* occurs at the damage formation rate (*k*_*4*_).

The tissue time derivatives (e.g., the rate of change of the tissue type with respect to time) can be expressed as a function of the rate constants (Supplementary Information 1; Eqs. S1-1 to S1-4). At steady-state, time derivatives are equal to zero which allows constant cycling rates (k_1_, k_2_, k_3_, k_4_, & k_5_) to be constructed from observable histological data.

Consistent with solutions of outflow closed systems, the steady-state volume solutions are underdetermined to a constant [Eqs. (S1-1) to (S1-7)]^29,30^. The constant can be eliminated if the system is written in terms of volume fractions [Eqs. (1b) and Eqs. (S1-8) to (S1-11)]. Therefore, the steady-state volume fractions equations [Eqs.(S1-8) to (S1-11)] will be used to solve for the steady state rate constants. However, the system is underdetermined when solving for five rate constants with four volume fractions. Therefore, we add an expression for tissue mineral density (TMD) to solve for the five rate constants. All model variables are defined in Table 1.

1b $$\frac{B{V}_{UD}}{TV}+\frac{B{V}_{D}}{TV}+\frac{OV}{TV}+\frac{MV}{TV}=1$$

### Dependence of tissue mineral density (TMD) on remodeling rates

TMD, defined by μCT, is the equivalent density of hydroxyapatite within a volume of mineralized tissue and changes with tissue age^31–33^. We express the average TMD using population statistics and model rate constants (Eq. 2)^34,35^. In Eq. (2), m(t) is a continuous bounded exponential growth function defining TMD during secondary mineralization (Eq. 3; see Supplementary Information 2 for derivation); primary mineralization is not considered because we assume primary mineralization has completed once *OV* transfers into *BV*_*UD*_. Further, P(t) is the probability distribution function describing the chances of mineralized bone resorption at time t^35^. We assume resorption of mineralized bone is independent of the amount of time spent in a compartment and use an exponential probability distribution for P(t) (Eq. (S2-1)).

2 $$TMD= {\int }_{0}^{\infty }m\left(t\right)P\left(t\right)dt$$

3 $$m\left(t\right)=\left(TM{D}_{o}-TM{D}_{max}\right){e}^{-{k}_{m}t} +TM{D}_{max}$$

TMD measurements by μCT do not distinguish between the TMD of *BV*_*D*_ and *BV*_*UD*_ [see Eqs. (S2-2) and (S2-3)], we need an equation to represent the average TMD within the entire mineralized bone volume (*BV*_*M*_/*TV*; Eq. 4). We calculate the average TMD of mineralized bone volume (*TMD*_*BVM*_) as weighted average of the TMD in *BV*_*D*_ and *BV*_*UD*_ (Eq. 5). Note that TMD_BVM_ depends on rate constant magnitude, while the steady-state volume fractions (Supplementary Information 1) depend on ratios of the rate constants.

4 $$\frac{B{V}_{M}}{TV}=\frac{B{V}_{UD}}{TV}+\frac{B{V}_{D}}{TV}$$

5 $$TM{D}_{B{V}_{M}}=\frac{\frac{B{V}_{UD}}{TV}}{\frac{B{V}_{M}}{TV}}TM{D}_{B{V}_{UD}}+\frac{\frac{B{V}_{D}}{TV}}{\frac{B{V}_{M}}{TV}}TM{D}_{B{V}_{D} }$$

### Relating observed morphometric data to model volume fractions and mineral density

Bone volume fraction (BVF), void volume fraction (1 – BVF), and TMD were measured via μCT^25^. BVF distinguishes mineralized bone from void using a mineralization threshold (540 mgHA/ccm). We assume *BV*_*M*_/*TV* is equal to the measured BVF (i.e., *BV*_*M*_/*TV* = BVF) and the unmineralized bone volume fraction (*BV*_*UM*_/*TV*; Eq. 6) is equal to the measured void volume fraction (i.e., *BV*_*UM*_/*TV* = 1 – BVF). The unmineralized bone volume fraction is determined using the measured BVF (i.e., *BV*_*UM*_/*TV* = 1 – BVF). We lacked data to distinguish *OV*/*TV* and *MV*/*TV*. Therefore, one rate constant (k_3_) was fixed among samples to solve for the remaining steady-state rate constants (Supplementary Information 3).

6 $$\frac{B{V}_{UM}}{TV}=\frac{MV}{TV}+\frac{OV}{TV}$$

Our BVF measurements cannot distinguish between BV_D_ and BV_UD_ (Fig. 1). Therefore, we defined *BV*_*D*_/*TV* as the area fraction of basic fuchsin stained linear microcracks (i.e., Cr.Af = ƩCrack Area/ROI Area)^36^. For each microcrack, stained crack area was defined as crack length^25^ multiplied by crack width. Crack width was defined as the basic fuchsin halo width measured at the microcrack midpoint (ImageJ)^37,38^. The area diffusely stained with basic fuchsin was not included in *BV*_*D*_/*TV*, as we did not determine if diffuse staining was due to diffuse microdamage or tissue with a low mineral density. *BV*_*UD*_/*TV* was determined by subtracting Cr.Af from the measured BVF (i.e., *BV*_*UD*_/*TV* = BVF − Cr.Af).

The TMD measured by µCT defines *TMD*_*o*_, *TMD*_*max*_ and each ROI’s TMD_BVM_. Due to our assumption that BV_M_ has completed primary mineralization, *TMD*_*o*_ is the value distinguishing primary and secondary mineralization. Therefore, *TMD*_*o*_ is the µCT mineralization threshold used to distinguish mineralized bone from non-mineralized tissue (540 mgHA/ccm; Table 1). Similarly, *TMD*_*max*_ is the average peak observed mineral density (1163.7 mgHA/ccm; Table 1; Fig. 2). The peak TMD of each PSB was determined from the whole PSB’s TMD histogram (Fig. 2) and was defined as 3.115 standard deviations from the histogram’s mean TMD^39^. In both ROIs, TMD_BVM_ equals the TMD measured in that ROI.

### Determining steady-state rate constants

Steady-state rate constants were determined in the Non-Damaged ROI and then in the Damaged ROI using the constants in Table 1 (Fig. 2). Supplementary Information 1 gives the two sets of steady-state volume fraction equations, written in terms of the rate constants, that were solved in the two ROIs. The equation sets are different in each ROI, because the Non-Damaged ROI has *BV*_*D*_/*TV* = 0 which requires *k*_*4*_ = *k*_*5*_ = 0 and the Damaged ROI has BV_D_/*TV* > 0, requiring k_4_, k_5_ > 0.

In the Non-Damaged ROI, *TMD*_*BVM*_ (Eq. 5) was solved directly for *k*_*1*_ using the measured TMD; this solution for *k*_*1*_ is independent of *k*_*3*_. Then, the determined *k*_*1*_ value and selected *k*_*3*_ were used in the steady state *BV*_*UD*_/*TV* equation, Eqs. (S1-19), to determine *k*_*2*_. The Non-Damaged ROI’s volume fraction solutions depend on the ratios k_1_/k_3_ and k_2_/k_3_ (Fig. S1-1).

The smallest feasible *k*_*3*_ (e.g., minimum k_3_ that allowed for *k*_*2*_ > 0) was determined in each Non-Damaged ROI and compared among Groups. This comparison was done to check if our assumption of a fixed *k*_*3*_ among all horses was reasonable given our data. To determine the effect of k_3_ on exercise regressions and model solutions, we solved for *k*_*2*_ with *k*_*3*_ = 1.024, 10, and 100 days^−1^. The minimum *k*_*3*_ that allowed for a *k*_*2*_ > 0 in all Non-Damaged ROIs was 1.024 days^−1^. The two larger *k*_*3*_ values (*k*_*3*_ = 10, 100) were chosen because *k*_*1*_ < 1 for all samples and large *k*_*3*_ values guarantee *k*_*1*_*/k*_*3*_ solutions within the *BV*_*M*_/*TV* range observed in the Non-Damaged ROI (see Fig. S1-1).

For each Damaged ROI, a non-linear least squares solver (MATLAB, lsqnonlin) was used to solve the steady-state *BV*_*UD*_/*TV* (Eq. S1-8), *BV*_*D*_/*TV* (Eq. S1-9), *BV*_*UM*_/*TV* (Eq. S1-12) and *TMD*_*BVM*_ (Eq. 5) for *k*_*1*_*, k*_*2*_*, k*_*4*_, and *k*_*5*_. The lower bound for the solutions vector was 0 to prevent the solver from returning negative rate constants. For each PSB, the solver was run with the starting points for k_4_ and k_5_ randomly varied between 0 and 100 and *k*_*1*_ and *k*_*2*_ set to Non-Damaged ROI’s steady-state solution; the solution vector from lsqnonlin (*k*_*1*_*, k*_*2*_*, k*_*4*_, and *k*_*5*_) returning the smallest root-mean-square error was selected. This process was performed with k_3_ = 1.024, 10, and 100 days^−1^. One CLI PSB had *BV*_*D*_/*TV* = 0 in the Damaged ROI; for this sample, rate constants were solved using the methods described for the Non-Damaged ROI.

### Racehorse exercise data

Official racing-speed activities were known for study horses (Jockey Club Information Systems Database)^17,25^. Exercise Events are either classified as a Race or Work (a high-speed training activity). A layup was defined as ≥ 60 days without an Event. Exercise was characterized for the entire career and during active training periods, which excluded time periods when horses were in a layup. Exercise data was used to derive 67 exercise variables in four categories: lifetime exercise, layup, exercise intensity, and exercise intensity in the year before death^25^.

### Statistical analysis

The relationships between rate constants and morphometric data were determined using Spearman correlation coefficients (r; SAS 9.4) in each ROI. The relationships between rate constants and the natural logarithm of the rate constants (ln(k_i_)) to exercise variables were determined using robust linear regressions with CTRL-PSB and CLI-PSB data (SAS 9.4; mm-method)^40^. A linear mixed model with horse as a random variable was performed to determine the effects of Group (FX, CLI, and CTRL) and ROI (Damaged, Non-Damaged) on the calculated rate constants (*k*_*1*_*, k*_*2*_*, k*_*4*_*, k*_*5*_) and morphometric data (BVF, TMD, Cr.Af; SAS 9.4; proc mixed). Ranked data were used to construct the linear mixed models when models built with raw data had residuals that were not normally distributed (W < 0.90). Comparisons of model means were performed with a Tukey–Kramer correction. In all analyses, p ≤ 0.05 was considered statistically significant.

Additional analyses were performed to check model assumptions. A linear mixed model, with horse as a random variable, was performed to determine if the smallest feasible *k*_*3*_ was different among Groups (CTRL, CLI, FX) within the Non-Damaged ROI (SAS 9.4; proc mixed). Additionally, the Borgonovo sensitivity of *k*_*1*_ to *k*_*m*_, *TMD*_*ROI*_, and *TMD*_*max*_ was determined for the Non-Damaged ROI (Supplementary Information 5)^41,42^.

## Results

### Tissue measures

TMD was significantly higher in the Non-Damaged ROI than in the Damaged ROI for all three groups. In FX-PSBs, BVF was lower in the Damaged ROI (90 ± 2%) than in the Non-Damaged ROI (96 ± 2%). The opposite relationship was seen in CTRL-PSBs, where BVF was higher in the Damaged ROI (98 ± 2%) than the Non-Damaged ROI (90 ± 2%). In the CLI-PSBs, no regional differences in BVF were observed (96 ± 2% Damaged ROI; 97 ± 2% Non-Damaged ROI). Cr.Af was higher in the Damage ROI than the Non-Damaged ROI for all groups. Cr.Af was higher in the FX-PSBs (0.020 ± 0.017 mm^2^/mm^2^) than in CLI-PSBs (0.008 ± 0.006 mm^2^/mm^2^) and CTRL-PSBs (0.004 ± 0.004 mm^2^/mm^2^). See Supplementary Information 4, Table S4-1, for all comparisons.

### Effect of k_3_

Group did not have a significant effect on the smallest feasible *k*_*3*_ in the Non-Damaged ROI. The choice of *k*_*3*_ had a limited effect on significant correlations and exercise regressions. Only *k*_*3*_ = 100 days^−1^ resulted in targeted damage removal in the Damaged ROI of all samples (i.e., *k*_*5*_ > *k*_*1*_). Therefore, all remaining results use *k*_*3*_ = 100 days^−1^. See Supplementary Information 3 for more details.

### Differences among rate constants

ROI had a significant effect on k_1_, k_4_ and k_5_ and, on average, these three rate constants were higher in the Damaged ROI than in the Non-Damaged ROI. ROI had a marginal effect on k_2_ (p = 0.07) and the interaction of ROI and Group had a significant effect on *k*_*2*_*.* CTRL-PSBs had a higher k_2_ in the Damaged ROI than the Non-Damaged ROI; in FX and CLI-PSBs, k_2_ was also higher in the Damaged ROI than Non-Damaged ROI, but the difference was not significant*.* In the Damaged ROI, *k*_*1*_ < *k*_*5*_ for all samples. Group did not have a significant effect on rate constants. Average rate constants are given in Supplementary Information 4 (Table S4-3).

Based on the mean steady-state rate constants and *k*_*3*_ = 100 days^−1^, the average time for one “volume unit” to complete the non-damaging model loop (i.e., sum of mean retention times)^34^ at steady-state was 187 days for FX-PSBs, 202 days for CLI-PSBs, and 195 days for CTRL-PSBs. The mean time for a volume unit to cycle through the full model is 151 days for FX-PSBs, 153 days for CLI-PSBs, and 161 days for CTRL-PSBs.

### Steady-State rate constants solutions based on morphometric data

**Figure 3:**
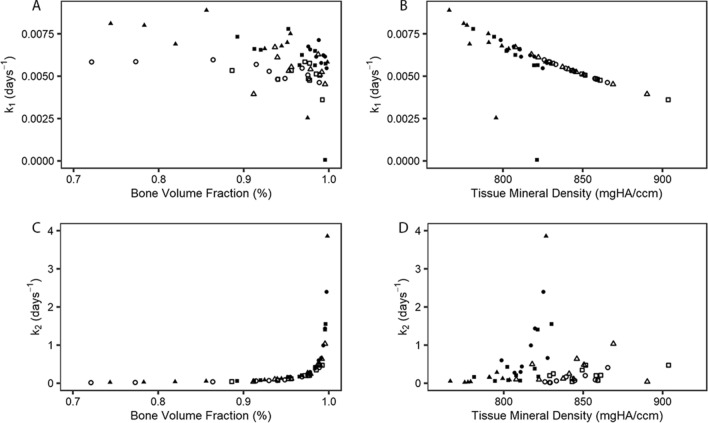
Non-damaged ROI (open symbols) and damaged ROI (filled symbols) solutions. (**A**) Undamaged resorption rate (*k*_*1*_) shown against measured bone volume fraction, (**B)** *k*_*1*_ shown against measured tissue mineral density, (**C)** osteoid formation rate (*k*_*2*_) shown against measured bone volume fraction, (**D)** *k*_*2*_ shown against measured tissue mineral density. In all panels, data from FX PSB (triangle) CLI PSBs (square) and CTRL PSBs (circles) are shown.

In both ROIs, BVF and TMD were negatively correlated with k_1_ and BVF was positively correlated with k_2_ (Fig. 3). TMD was not correlated with k_2_ in the Non-Damaged ROI, but was positively correlated with k_2_ the Damaged ROI (r = 0.81; Fig. 3). In the Non-Damaged ROI, the k_1_ and k_2_ solutions were not correlated; in the Damaged ROI, k_1_ was negatively correlated with k_2_ (r = −0.74).

**Figure 4:**
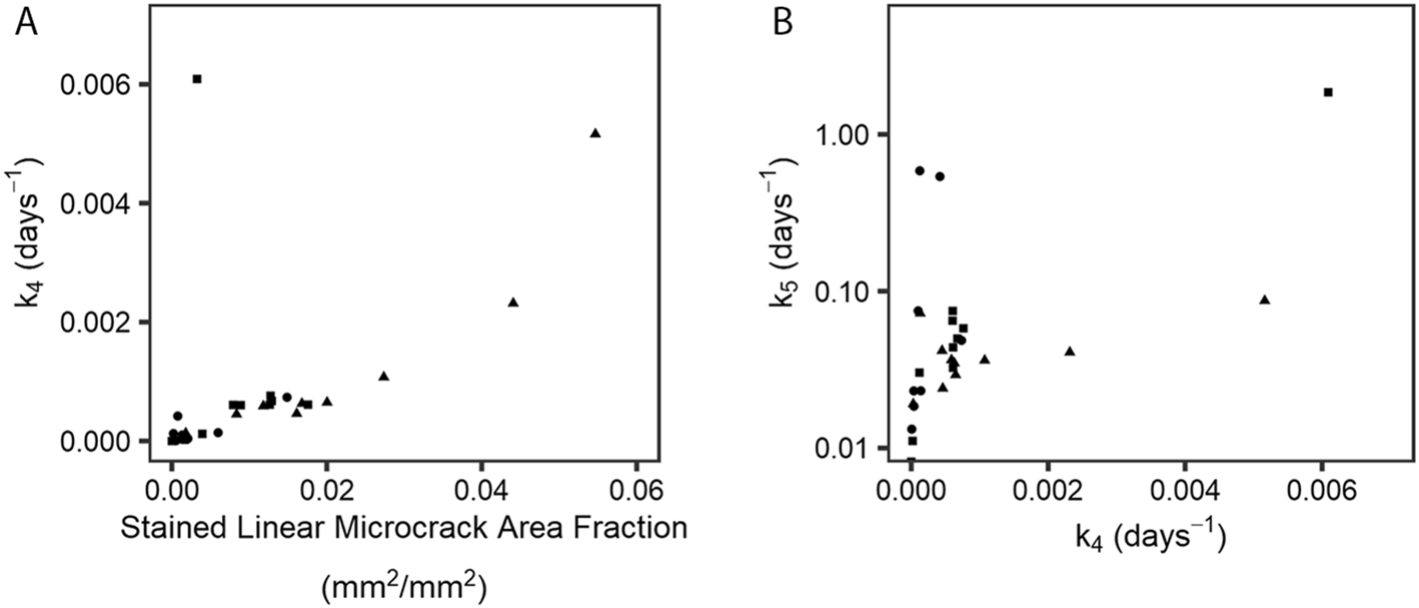
Damaged ROI solutions for the damage formation rate (*k*_*4*_) and damage resorption rate (*k*_*5*_) shown for the FX PSBs (triangle), CLI PSBs (square) and CTRL PSBs (circle). (**A)** *k*_*4*_ plotted against the stained microcrack area fraction (Cr.Af); note, in the model, *BV*_*D*_/*TV* ≡ Cr.Af. (**B)** *k*_*4*_ plotted against *k*_*5*_*.* In both panels, solutions for the non-damaged ROI are not shown, as Cr.Af, *k*_*4*,_ and *k*_*5*_ were zero in the non-damaged ROI.

In the Damaged ROI, Cr.Af was positively correlated with k_4_ (r = 0.82), but Cr.Af had no relationship to k_5_ (Fig. 4). Also, k_4_ was not correlated with k_1_, k_2_, BVF, or TMD, but was positively correlated to k_5_ (r = 0.54). Further, k_5_ was correlated with k_1_ (r = −0.64), k_2_ (r = 0.39), BVF (r = 0.38), and TMD (r = 0.37). All correlations are tabulated in Supplementary Information 4, Table S4-2.

### Regressions between exercise data and steady-state rate constants

The observed relationships between exercise, steady-state rate, and morphometric data^25^ are summarized in Table 2. All significant regressions are given in Supplementary Information 4.

**Table 2:** Summary of how the steady-state rate constants, mineralized bone volume fraction, and tissue mineral density changed with exercise intensity in the two regions of interest (ROI).

	Resorption rate (*k*_*1*_)	Osteoid formation rate (*k*_*2*_)	Damage formation rate (k_4_)	Damage resorption rate (k_5_)	BVF	TMD
Damaged ROI	NS	↓ exercise	↑ exercise	NS	↓ exercise	↓ exercise
Non-Damaged ROI	↑ exercise	↑ exercise	N/A	N/A	↑ exercise	↓ exercise

NS indicates no significant relationships were observed and N/A indicates the relationships were not calculated. The relationships between BVF and TMD in the two ROIs is described in Shaffer et al., 2022. Note: primary mineralization rate (*k*_*3*_) was fixed.

**Figure 5:**
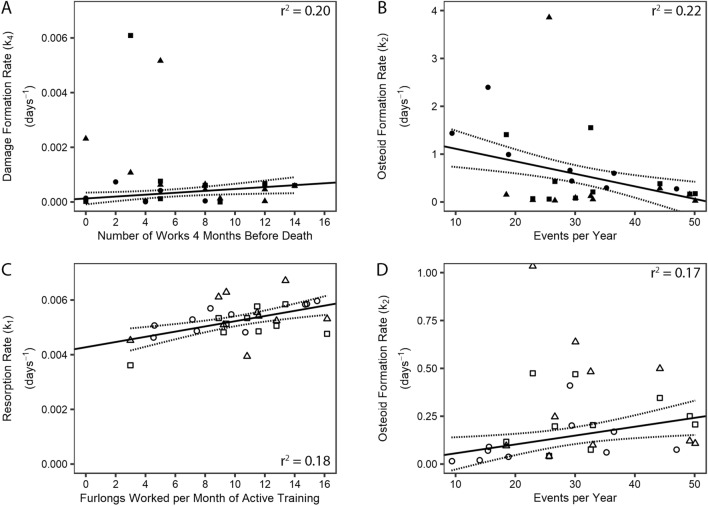
Significant robust linear regressions between *k1, k2, k4* and exercise history variables in the damaged ROI (**A,B**) and non-damaged ROI (**C**,**D**); the 95% confidence intervals (dashed lines) are shown. All regressions were made using CTRL PSB (circle) and CLI PSB (square) data. The direction of increasing exercise intensity is shown by the arrow along the exercise axis. Data from FX PSB (triangle) PSBs were not used to construct the robust regressions; however, when CTRL & FX data were used to construct the regressions, the directions of the relationships shown stayed the same.

The resorption rate (*k*_*1*_) generally increased with exercise intensity in the Non-Damaged ROI (Table 2). For example, *k*_*1*_ increased with exercise intensity during active training periods (r^2^ = 0.14–0.23; Fig. 5C). However, *k*_*1*_ decreased with the number of races 10 months before death (r^2^ = 0.11). Exercise variables were more strongly related to *k*_*1*_, rather than ln(*k*_*1*_), in the Non-Damaged ROI. In the Damaged ROI, there were no significant relationships between exercise and *k*_*1*_ (Table S4-4).

The relationships between osteoid formation rate (*k*_*2*_) and exercise history had different directions in the Damaged and Non-Damaged ROIs (Fig. 5B,D and Table 2; Table S4-5). In the Non-Damaged ROI, *k*_*2*_ increased with cumulative races 1–10 months before death (r^2^ = 0.17–0.23), increased with career exercise intensity variables (r^2^ = 0.17–0.20), and decreased with time in layup (r^2^ = 0.17–0.27). However, *k*_*2*_ did increase in the Non-Damaged ROI with days between works during active training. In the Damaged ROI, *k*_*2*_ decreased with cumulative high-speed exercise 1–12 months before death (r^2^ = 0.11–0.38), career exercise intensity (r^2^ = 0.15–0.22), and the number of days and events since the last layup (r^2^ = 0.12–0.15). In the Damaged ROI, *k*_*2*_ increased with time in layup (r^2^ = 0.23). In both ROIs, ln(k_2_) was more strongly related to exercise variables than k_2_ (Table S4-5).

In the Damaged ROI, the damage formation rate (*k*_*4*_) increased with the high-speed workouts 4 months before death (r^2^ = 0.20; Fig. 5A) and ln(k_4_) increased with time between races during active training periods (r^2^ = 0.19) and average layup length (r^2^ = 0.13; Table S4-6). No relationships were observed between damage resorption rate (*k*_*5*_) or ln(*k*_*5*_) and exercise.

## Discussion

This study derived steady-state rate constants (k_1_, k_2_, k_3_, k_4_, and k_5_) for the compartment model of bone’s tissue turnover cycle (Fig. 1) using morphometric data, collected post-mortem, from racehorse PSBs. Rate constants were determined for two ROIs: a subchondral location with low bone density and high levels of microdamage (Damaged ROI) and in an internal region with no microdamage (Non-Damaged ROI).

The correlations between BVF and TMD with resorption rate (*k*_*1*_) and osteoid formation rate (*k*_*2*_) indicate the solving procedure predicted steady-state rate constants from morphometric data that are consistent with model equations. The negative correlations observed between *k*_*1*_ and BVF (BVF = *BV*_*M*_/*TV*), in both ROIs is consistent with the partial derivative of *BV*_*M*_/*TV* with respect to k_1_, which is always less than zero (Eq. S1-14). Similarly, positive correlations between *k*_*2*_ and BVF in both ROIs are consistent with the partial derivative of *BV*_*M*_/*TV* with respect to k_2_, which is always greater than zero (see Eq. S1-14). The steady-state damage resorption rates (k_5_) were greater than the undamaged resorption rate (k_1_) within the Damaged ROI with the choice of k_3_ used to solve the equations. This observation is consistent with targeted remodeling because the average time to resorb damaged tissue (1/*k*_*5*_) is faster than for undamaged tissue (1/k_1_) if *k*_*5*_ > k_1_. If *k*_*5*_ > *k*_*1*_, an increase in k_4_ would decrease *BV*_*M*_/*TV* (see Eq. S1-17); however, we did not see a significant correlation between k_4_ and BVF.

Rate constants were found under the assumption of steady state (e.g., unchanging rates of bone formation, resorption, etc.). Living bone has changing turnover rates affected by load, age, sex, medication, and other factors^10^. As a result, the steady-state assumption includes the supposition that the horse activity levels have gone on long enough that all turnover rates have reached equilibrium, which is a limitation of this study. However, since bone’s turnover cycle is affected by strain magnitude, strain rate, changes in loading conditions from a habitual condition and other factors^10^, both the dynamic and steady-state rate constants are expected to be related to exercise (e.g., applied load). Exercise intensity, layups, and exercise before death variables probably represent recent strain rate (or changes in strain rate) better than lifetime exercise variables. Therefore, it is not surprising that these three variable types had stronger relationships to *k*_*1*_, *k*_*2*_, and *k*_*4*_ compared to lifetime exercise variables for model solutions that assume steady-state turnover at the time of death.

Bone damage removal is likely targeted^9,43^. However, damage removal could be modified by the amount of exercise or the amount of damage present in the tissue. Our results do not support exercise modifying the damage resorption rate (*k*_*5*_), since we found no relationships between *k*_*5*_ and exercise. However, *k*_*5*_ was positively correlated to the damage formation rate (*k*_*4*_), which suggests the rate of damage formation directly effects the rate of damage repair. This correlation was found under the assumption of steady-state, so, future dynamic turnover models should test the hypothesis that *k*_*5*_ depends on the amount of tissue damage. A damage repair rate (k_5_) that increases with the amount of damage is consistent with observations in fracture repair and is implicated in studies of microdamage-related osteocyte apoptosis^44^. Further, it is supported by observations that in fatigue-loaded bone, activation of resorption depends on the presence of linear microcracks but not the duration of loading^43^.

The relationships between resorption rate (*k*_*1*_) and osteoid formation rate (*k*_*2*_) with exercise history had different directions in the Damaged and Non-Damaged ROIs (Table 2; Supplementary Information Tables S14-4 and S14-5). These regional differences in the exercise relationships are consistent with damaging loading (i.e., high strain) consistently reducing bone formation and/or increasing bone resorption within the subchondral tissue (Damaged ROI) but not within the deeper tissue (Non-Damaged ROI). In the Damaged ROI, *k*_*2*_* decreased* with exercise intensity. In the Non-Damaged region, both *k*_*1*_ and *k*_*2*_* increased* with exercise intensity. Previously we observed that BVF *decreased* with exercise intensity in the Damaged ROI and *increased* with exercise intensity in the Non-Damaged ROI (Table 2)^25^. Model equations indicate a decrease in BV_M_/*TV* (the measured BVF) is associated with a decrease in k_2_ and an increase in k_1_ (Eqs. S1-15 and S1-16) and, if *k*_*5*_ > *k*_*1*_, an increase in *k*_*4*_ (Eq. S1-17). These predictions are consistent with the relationships observed between the model rate constants, BVF, and exercise if a shared exercise-related factor affected *k*_*2*,_ and *k*_*4*_ differently in the two regions. Further, an increase in *k*_*1*_ with exercise intensity in the Non-Damaged ROI is consistent with a shared exercise-related factor if the factor affects *k*_*2*_ more than k_1_ (Eqs. S1-15 and S1-16) or if the cycle rate increases with exercise. We previously observed that TMD decreased with exercise frequency (implying the cycle rate increases with exercise intensity), but that TMD in the Non-Damaged ROI was similar among all horses^25^. Therefore, we expect the exercise-related factor to affect *k*_*2*_ more than *k*_*1*_; however, a dynamic simulation would be useful in testing this hypothesis.

We hypothesize that the exercise related factor that drives the rate constants in different directions in the two regions is strain. Both ROIs experienced the same exercise intensity (because they were in the same bone of the same horse), however, there will be a mechanical strain difference between subchondral tissue (Damaged ROI) and tissue deeper to a joint surface (Non-Damaged ROI). Strain is a known driver of damage formation and bone’s response to load^10^. So, a strain difference between the two regions is consistent with an exercise-related factor driving *BV*_*M*_/*TV*, *k*_*1*_, k_2_ in opposite directions in the two regions. Other factors, besides a difference in strain states, could also impact the model rate constants.

Bone turnover that favors increased formation and decreased resorption has been proposed to occur in the third metacarpal bone (MC3) of racehorses due to intense training^45^. For example, less eroded (resorption) surface, more microdamage, and more osteoid was observed at a common subchondral stress-remodeling site in MC3 condyles of racehorses in training compared to resting racehorses^46^. However, in the same study, the amount of erosion surface in active horses was positively associated with time in training^46^; this finding is consistent with our results in the Damaged ROI if k_5_ (damaged bone resorption) is driven by the damage formation rate (k_4_) or the presence of damage (both of which increased with exercise in our study). Similarly, the amount of eroded surface and mineralizing surface was higher near an MC3 fatigue fracture site in active racehorses that sustained MC3 fracture compared to active racing controls^47^; these findings also imply increased in remodeling near a fatigue-fracture sites, consistent with our results in the Damaged ROI. Also, Damaged ROI TMD was negatively correlated to exercise, indicating younger tissue or more newly deposited tissue in that region^25^. However, bone’s acute and long-term responses to exercise remains unclear^48,49^. A dynamic simulation, with variable rate constants, would be needed to assess the effect of altering bone formation and turnover balance on tissue volume fractions—but is beyond the scope of this manuscript.

Damage formation rate (*k*_*4*_) increased with the number of racing-speed workouts 4 months before death and lifetime works (Supplementary Information Table S14-6). We observed that *k*_*4*_ was more strongly related to exercise 4 months before death (r^2^ = 0.20) than it was to any other variable. Further, our previous work that indicates microcrack number and areal microcrack density increased with workouts 4 months prior to death (r^2^ = 0.18, 0.29) in the Damaged ROI^25^. Additionally, BVF was observed to decrease with cumulative exercise 2–10 months prior to death in the Damaged ROI^25^. From these observations, we hypothesize that damage formation rate is more strongly related to recent loading history (< 6 months) rather than to lifetime exercise. A clinically important observation is that exercise over a period of 4–6 months is a good candidate for management by trainers that could prevent formation of the subchondral lesion. This observation is consistent with previous epidemiological work that demonstrates that recent exercise activity is associated with racehorse stress-fractures^50–52^.

In fatigue-testing and computational stress-analysis, damage is often defined as a material modulus reduction. Many types of damage are observed in bone tissue and are associated with modulus reduction^6,53^. We defined the damaged mineralized volume fraction (*BV*_*D*_/*TV*) as the stained linear microcrack area fraction. However, many histology sections contained bone tissue diffusely stained with basic fuchsin^25^, which denotes the presence of low TMD and/or diffuse microdamage^53,54^. We do not know what the diffuse stain represents in these sections, however, both low TMD and diffuse damage imply a reduced modulus in an area with diffuse stain compared to unstained tissue^5,6^. We estimated crack width using the basic fuchsin stained halo (mean width ~ 14 μm), which is larger than microcrack widths reported in literature (~ 4 μm)^55^. Therefore, while our crack width definition may not reflect a physical microcrack width, the *BV*_*D*_/*TV* estimate represents a damaged region with reduced modulus compared to undamaged tissue in a consistent manner. Further, cracks in the calcified cartilage layer often extended into the subchondral tissue were identified in Case bones^25^. These cracks would reduce subchondral tissue modulus; however, calcified cartilage cracks were not included in the BV_D_/*TV* because calcified cartilage cracks are not repaired by remodeling^56^. Finally, fatigue-loading models have shown bone resorption is associated with linear microcracks and suggest diffuse damage repair can occur without remodeling^43^. So, our use of linear microcracks to estimate a damage volume fraction in the compartment model is consistent with observable tissue repair processes.

We assumed bone exiting the osteoid compartment had completed primary mineralization. Generally, primary mineralization is considered the accumulation of 0–70% of maximum possible mineral density and secondary mineralization accounts for 70–95% of maximum possible mineral density^3^. However, the threshold distinguishing primary and secondary mineralization phases is sensitive to both measurement resolution and the definition of mineral content. For example, older estimates for the percentage of mineralization completed during primary mineralization (70%) did not track mineralization changes over time and have a lower resolution compared to μCT^3,57^. A wider range of minimum-to-maximum mineralization density (~ 30–70%) is reported in studies performed at a higher resolution using radiodensity referenced to hydroxyapatite to define mineral content^58–62^. The average minimum-to-maximum TMD ratio for our samples was 46.4%, which is within the range reported in more recent studies. Additionally, bone mineral is not purely hydroxyapatite, but instead contains a wide variety of anionic and cationic substitutions in the hydroxyapatite lattice and the mineralization rate of chemical species occurs at different rates in bone tissue^63,64^. So, it is possible that traditional measurements do not fully capture the primary and secondary mineralization processes. Therefore, while our threshold distinguishing primary and secondary mineralization (46.4%) is lower than the traditional 70% value, we think using the observable threshold from µCT is an appropriate method to separate the two mineralization regimes.

Study limitations include the sample size (20 horses; 30 PSBs) and the lack of osteoid data. The sample size makes the statistical power of this study low, similar results with additional racehorse PSB data or from a study of another stress fracture site would increase confidence in the findings. However, the comparison of damaged and non-damaged regions within the same horse may have reduced variation due to individual differences and helped identify relationships within this sample set. Because we did not measure osteoid, we set the mineralization rate (*k*_*3*_). However, *k*_*3*_ is unknown in horses and there is limited evidence it is affected by mechanical loading (unlike the other model rate constants), it was a good candidate to assume fixed among horses. Our results indicate the choice of *k*_*3*_ did not affect correlations with morphometric measures or the relationships among rate constants and exercise.

In summary, we determined steady state rate constants for a compartment model of bone’s tissue turnover cycle using observed morphological data collected post-mortem from racehorse PSBs. We found significant relationships between the calculated steady-state rate constants and exercise. These relationships were consistent with bone biology and could be used to dynamically drive the rate constants with strain (or another exercise-related parameter) in a dynamic model.

## Supplementary Information

Supplementary Information.

## Data Availability

The datasets generated during and/or analyzed during the current study are available from the corresponding author on reasonable request.
