# Supplementary material for: Training drives turnover rates in racehorse proximal sesamoid bones
Source: Sci Rep. 2023 Jan 27;13:205. doi: 10.1038/s41598-022-26027-y (PMC9883508; doi:10.1038/s41598-022-26027-y)
Supplement: Supplementary file 1 — Supplementary Information. [file 41598_2022_26027_MOESM1_ESM.docx]

**Supplementary Information 1: Equilibrium Solution for Bone Compartment Model**

This supplemental information presents the equations for the compartment model of bone turnover given in Fig. 1. In this model, the 4 tissue types are: undamaged mineralized bone (*BV_UD_*), damaged mineralized bone (*BV_D_*), osteoid (*OV*), and marrow (*MV*). The time derivatives of these 4 tissue types can be expressed using the rate constants (k_i_) and are given in equations (S1-1 to S1-4). The summation of the 4 tissue types is equal to the total tissue volume (*TV*). To prevent negative volume components, all rates terms (k_i_) are non-negative.

| $\frac{d\left( BV_{UD} \right)}{dt} =k_{3}OV-\left( k_{1}+k_{4} \right)BV_{UD}$ | (S1-1) |
| --- | --- |
| $\frac{d\left( BV_{D} \right)}{dt}=k_{4}BV_{UD}-k_{5}BV_{D}$ | (S1-2) |
| $\frac{d\left( OV \right)}{dt} =k_{2}MV-k_{3}OV$ | (S1-3) |
| $\frac{d\left( MV \right)}{dt} =k_{5}DV+k_{1}BV_{UD}-k_{2}OV$ | (S1-4) |

At steady-state, the time derivatives are equal to zero. In matrix form, the steady-state equations are represented as:

$\left[ \begin{aligned} dBV_{UD}/dt \\ dMV/dt \\ dOV/dt \\ dBV_{D}/dt \end{aligned} \right]=\boldsymbol{M}*\boldsymbol{X}=\left[ \begin{matrix} -k_{1}-k_{4} & 0 & k_{3} & 0 \\ k_{1} & -k_{2} & 0 & k_{5} \\ 0 & k_{2} & -k_{3} & 0 \\ k_{4} & 0 & 0 & -k_{5} \end{matrix} \right]*\left[ \begin{aligned} BV_{UD} \\ MV \\ OV \\ BV_{D} \end{aligned} \right]= \left[ \begin{aligned} 0 \\ 0 \\ 0 \\ 0 \end{aligned} \right]$ (S1-5)

The general solution for the four tissue types is found using the null space of **M**. The basis vector for this null space is **X’** (equation (S1-6)). The general solution is, therefore, any constant (*B*) multiplied by **X’**.

**X’**=$\left[ \begin{aligned} \frac{k_{5}}{k_{4}} \\ \frac{k_{5} \left( k_{1}+k_{4} \right)}{k_{2} k_{4}} \\ \frac{k_{5} \left( k_{1}+k_{4} \right)}{k_{3} k_{4}} \\ 1 \end{aligned} \right]$ (S1-6)

However, the general solution to the system of equations (Eqn. S1-5) is subjected to the constraints that: *k_i_* > 0 and *BV_UD_* + *BV_D_* + *OV* + *MV* = *TV* (Eqn. 1). This limits the general solution to Eqn. S1-7a, where B is a constant that is greater than or equal to zero, and requires that *B***TV* > 0 (Eqn 7b). This solution indicates that 1) the volumes are related to ratios of the rate constants and 2) the total tissue volume. However, the solution is indeterminate to the constant *B*.

$\boldsymbol{X}= \left[ \begin{aligned} \mathrm{BV}BV_{UD} \\ MV \\ OV \\ BV_{D} \end{aligned} \right]=B*\left[ \begin{aligned} \frac{k_{5}}{k_{4}} \\ \frac{k_{5} \left( k_{1}+k_{4} \right)}{k_{2} k_{4}} \\ \frac{k_{5} \left( k_{1}+k_{4} \right)}{k_{3} k_{4}} \\ 1 \end{aligned} \right]$where B > 0, k_i_ > 0 (S1-7a)

$B*\left( BV_{UD}+BV_{D}+OV+MV \right)=TV>0$ (S1-7b)

To eliminate the unknown constant, *B*, from the equations, we will divide the general solution for the volumes (equation (S1-7a)) by the tissue volume (equation (S1-7b)). This results in four volume fractions that are independent of the constant *B* and that only depend on the *ratios* of the rate constants (equations (S1-8) to (S1-11)). These solutions hold for regions that contain damaged mineralized bone (i.e., within the Damaged ROI where *BV_D_*/*TV* > 0). The steady-state solutions for a region with no damage (i.e., the Non-Damaged ROI) are given in equations (S1-19) to (S1-25).

$\frac{BV_{UD}}{TV}= \frac{k_{2} k_{3} k_{5}}{k_{1}k_{2}k_{5}+k_{1}k_{3}k_{5}+k_{2}k_{3}k_{4} +k_{2}k_{3}k_{5} +k_{2}k_{4}k_{5} +k_{3}k_{4}k_{5}}$ (S1-8)

$\frac{BV_{D}}{TV}= \frac{k_{2} k_{3} k_{4}}{k_{1}k_{2}k_{5}+k_{1}k_{3}k_{5}+k_{2}k_{3}k_{4} +k_{2}k_{3}k_{5} +k_{2}k_{4}k_{5} +k_{3}k_{4}k_{5}}$ (S1-9)

$\frac{OV}{TV}=\frac{k_{2} k_{5} \left( k_{1}+k_{4} \right)}{k_{1}k_{2}k_{5}+k_{1}k_{3}k_{5}+k_{2}k_{3}k_{4} +k_{2}k_{3}k_{5} +k_{2}k_{4}k_{5} +k_{3}k_{4}k_{5}}$ (S1-10)

$\frac{MV}{TV}=\frac{k_{3} k_{5} \left( k_{1}+k_{4} \right)}{k_{1}k_{2}k_{5}+k_{1}k_{3}k_{5}+k_{2}k_{3}k_{4} +k_{2}k_{3}k_{5} +k_{2}k_{4}k_{5} +k_{3}k_{4}k_{5}}$ (S1-11)

The steady-state mineralized bone volume fraction (Eqn. S1-12) and unmineralized volume fraction (Eqn. S1-13) are as follows:

$\frac{BV_{UM}}{TV}= \frac{k_{5}(k_{2}+k_{3}) \left( k_{1}+k_{4} \right)}{k_{1}k_{2}k_{5}+k_{1}k_{3}k_{5}+k_{2}k_{3}k_{4} +k_{2}k_{3}k_{5} +k_{2}k_{4}k_{5} +k_{3}k_{4}k_{5}}$ (S1-12)

$\frac{BV_{M}}{TV}= \frac{k_{2} k_{3} {(k}_{5}+k_{4})}{k_{1}k_{2}k_{5}+k_{1}k_{3}k_{5}+k_{2}k_{3}k_{4} +k_{2}k_{3}k_{5} +k_{2}k_{4}k_{5} +k_{3}k_{4}k_{5}}$ (S1-13)

The partial derivatives of the mineralized bone volume fraction, equation (S1-13), with respect to the rate constants are given below (equations (S1-14) to (S1-18)).

$\frac{\partial\left( \frac{BV_{M}}{TV} \right)}{\partial k_{1}}= -\frac{k_{2} k_{3} k_{5} \left( k_{2}+k_{3} \right) \left( k_{4}+k_{5} \right)}{\left( k_{1}k_{2}k_{5}+k_{1}k_{3}k_{5}+k_{2}k_{3}k_{4} +k_{2}k_{3}k_{5} +k_{2}k_{4}k_{5} +k_{3}k_{4}k_{5} \right)^{2}}$ (S1-14)

$\frac{\partial\frac{BV_{M}}{TV}}{\partial k_{2}}= \frac{{k_{3}}^{2} k_{5} \left( k_{1}+k_{4} \right) \left( k_{4}+k_{5} \right)}{\left( k_{1}k_{2}k_{5}+k_{1}k_{3}k_{5}+k_{2}k_{3}k_{4} +k_{2}k_{3}k_{5} +k_{2}k_{4}k_{5} +k_{3}k_{4}k_{5} \right)^{2}}$ (S1-15)

$\frac{\partial\frac{BV_{M}}{TV}}{\partial k_{3}}=\frac{{k_{2}}^{2} k_{5} \left( k_{1}+k_{4} \right) \left( k_{4}+k_{5} \right)}{\left( k_{1}k_{2}k_{5}+k_{1}k_{3}k_{5}+k_{2}k_{3}k_{4} +k_{2}k_{3}k_{5} +k_{2}k_{4}k_{5} +k_{3}k_{4}k_{5} \right)^{2}}$ (S1-16)

$\frac{\partial\left( \frac{BV_{M}}{TV} \right)}{\partial k_{4}}=\frac{k_{2} k_{3} k_{5} \left( k_{2}+k_{3} \right) \left( k_{1}-k_{5} \right)}{\left( k_{1}k_{2}k_{5}+k_{1}k_{3}k_{5}+k_{2}k_{3}k_{4} +k_{2}k_{3}k_{5} +k_{2}k_{4}k_{5} +k_{3}k_{4}k_{5} \right)^{2}}$ (S1-17)

$\frac{\partial\left( \frac{BV_{M}}{TV} \right)}{\partial k_{5}}= -\frac{k_{2} k_{3} k_{4} \left( k_{1}+k_{4} \right) \left( k_{2}+k_{3} \right)}{\left( k_{1}k_{2}k_{5}+k_{1}k_{3}k_{5}+k_{2}k_{3}k_{4} +k_{2}k_{3}k_{5} +k_{2}k_{4}k_{5} +k_{3}k_{4}k_{5} \right)^{2}}$ (S1-18)

If no damage is present in a region (i.e., within the Non-Damaged ROI), then the damaged mineralized bone compartment contains no tissue. At steady state, this indicates that both *k_4_* and *k_5_* are equal to zero. The steady-state solution for the volume fractions is then equations (S1-19) to (S1-21); solution surfaces are shown in Figure S1-1. The steady-state equation for unmineralized bone volume fraction in a region without damage is shown in equation (S1-22).

$\frac{BV_{UD}}{TV}= \frac{k_{2} k_{3}}{k_{1} k_{2}+k_{1} k_{3}+k_{2} k_{3}}$ (S1-19)

$\frac{OV}{TV}= \frac{k_{1}k_{2}}{k_{1} k_{2}+k_{1} k_{3}+k_{2} k_{3}}$ (S1-20)

$\frac{MV}{TV}=\frac{k_{1} k_{3}}{k_{1} k_{2}+k_{1} k_{3}+k_{2} k_{3}}$ (S1-21)

$\frac{BV_{UM}}{TV}=\frac{k_{1} k_{2}+k_{1}k_{3}}{k_{1} k_{2}+k_{1} k_{3}+k_{2} k_{3}}$ (S1-22)


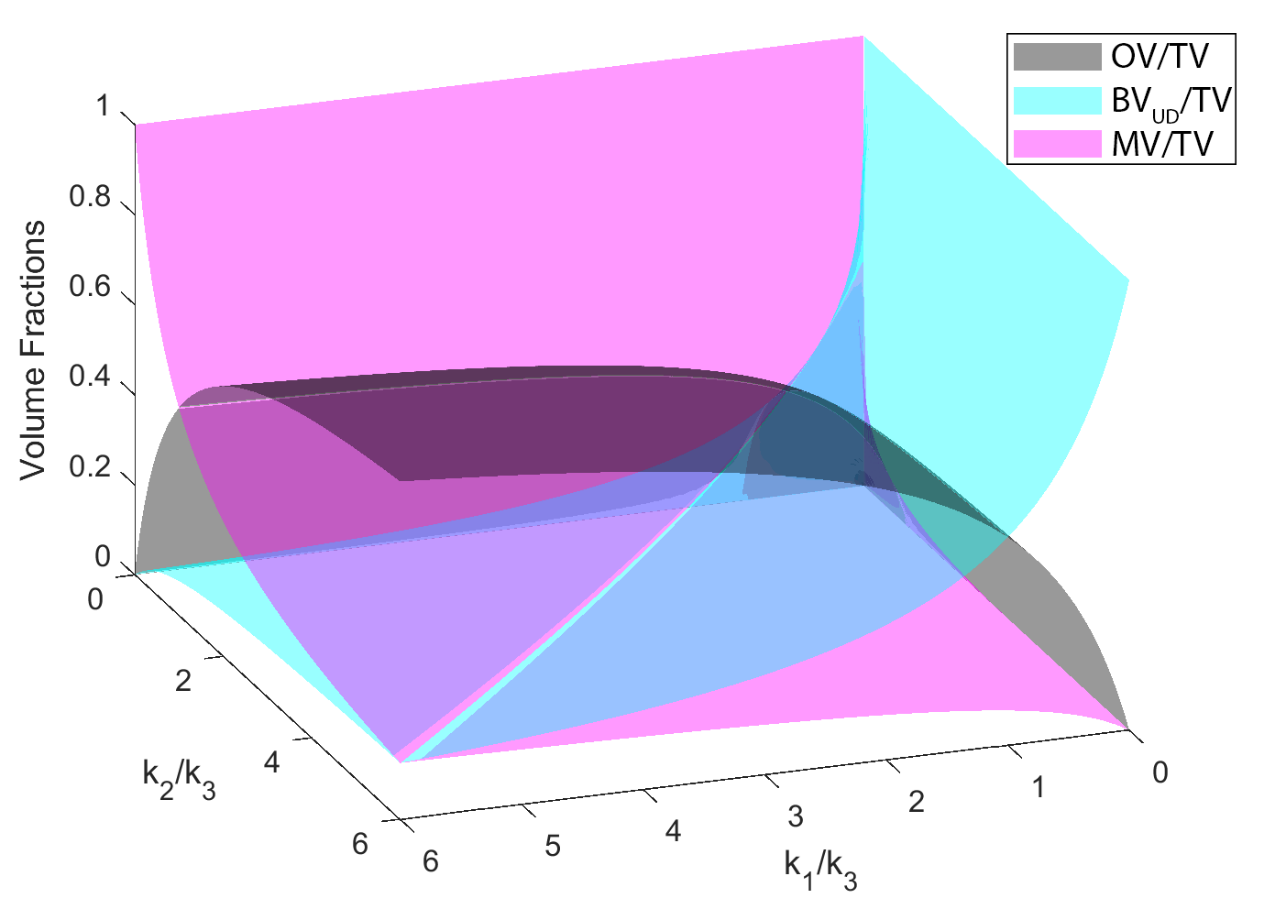


**Fig S1-1** Steady-state volume fraction surfaces for the Non-Damaged ROI. In the Non-Damaged ROI, BV_D_/TV = 0, so the measured bone volume fraction (BVF) is equal to the model’s undamaged mineralized bone volume fraction (BV_UD_/TV; blue surface).

The partial derivatives of the undamaged mineralized bone volume fraction in a region without damaged (equation (S1-19)) with respect to the steady state rate constants are shown in equations (S1-23) to (S1-25).

$\frac{\partial BV_{UD}}{\partial k_{1}}= -\frac{k_{2}k_{3}\left( k_{2}+k_{3} \right)}{\left( k_{1}k_{2}+k_{1}k_{3}+k_{2}k_{3} \right)^{2}}$ (S1-23)

$\frac{\partial BV_{UD}}{\partial k_{2}}= \frac{k_{1}k_{3}^{2}}{\left( k_{1}k_{2}+k_{1}k_{3}+k_{2}k_{3} \right)^{2}}$ (S1-24)

$\frac{\partial BV_{UD}}{\partial k_{3}}= \frac{k_{1}k_{2}^{2}}{\left( k_{1}k_{2}+k_{1}k_{3}+k_{2}k_{3} \right)^{2}}$ (S1-25)

**Supplementary Information 2: Tissue Mineral Density Derivation**

To use equation (2) to determine the tissue mineral density (TMD), we require a mineralization law, m(t), for the two mineralized bone compartments. Additionally, to use equation (2), the probability of resorption at a given time must be determined to create the probability distribution function, P(t).

***Mineralization Law***

We tried several forms for the mineralization function, m(t), including: a bounded exponential, the sum of two exponentials, a logistic, and a hyperbolic.^65^ All four forms had similar r^2^ values when curve-fit to secondary mineralization data from rabbits and sheep.^1,2^ For simplicity, we will model m(t) as bounded exponential growth (equation (3); Fig. S2-1). Previous work indicates that using the sum of two exponentials to define m(t), rather than a single exponential better reproduces the calcium concentration distribution (BMDD) observed in bone tissue. ^65^ However, since we are only using Eqn. 3 to determine average TMD, and not BMDD, we will use the simpler, single-exponential model.

**
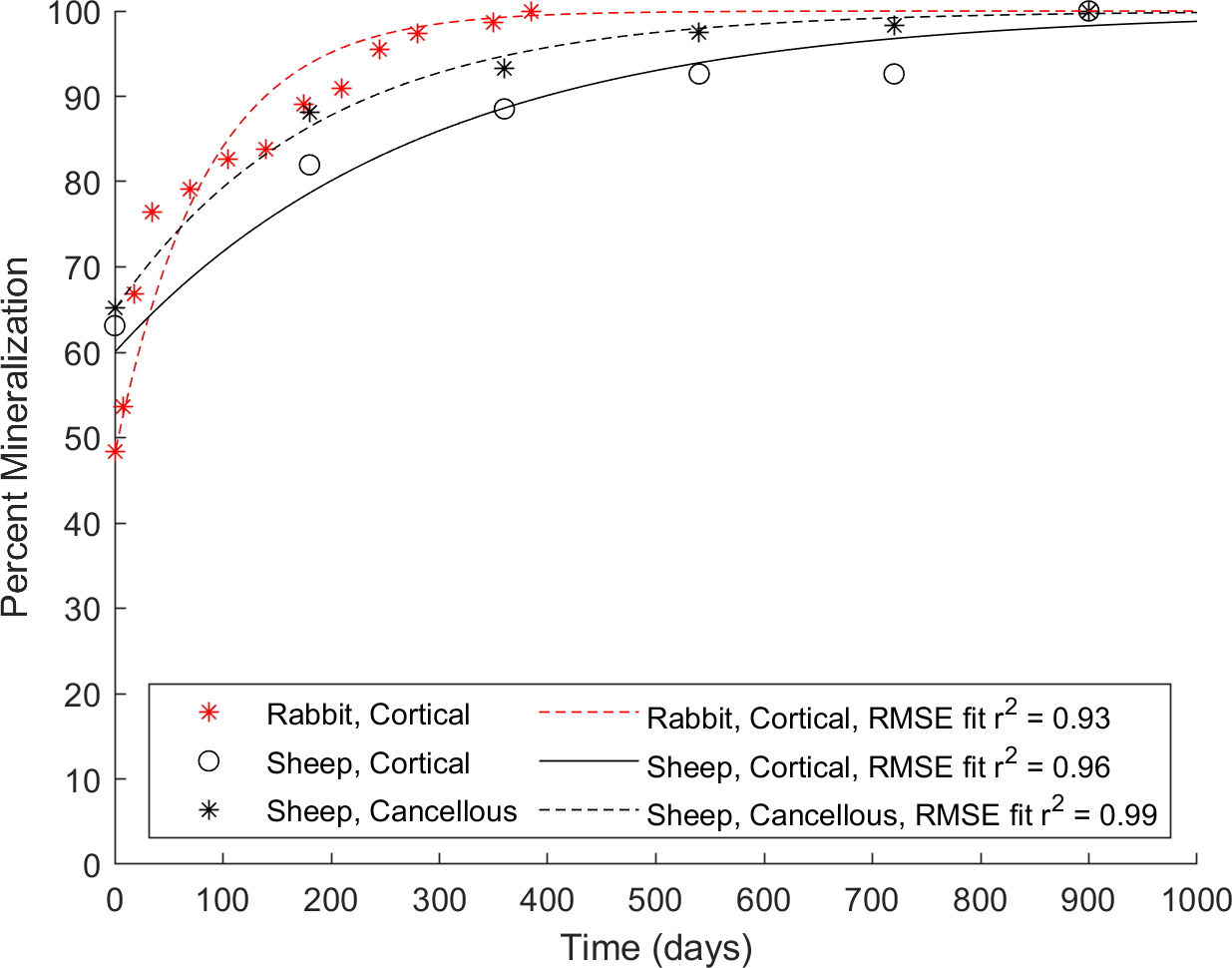
**

**Figure S2-1:** RMSE curve-fits of a bounded exponential (equation (3)) from rabbit and sheep bones. Data used for curve fits were digitized from Fuchs, et. al., 2008 (rabbit) and Bala, et. al., 2010 (sheep). Mineral content data was normalized to peak measured mineralization to create this figure; *TMD_o_* was the normalized measurement at the first time point and *TMD_max_* was the normalized peak measurement (i.e., 100%). When data was not normalized, the r^2^ values were similar and < 0.93. Curve-fits were performed in MATLAB’s non-linear least-square’s solver using the Levenberg-Marquardt option.

The secondary mineralization rate constant (*k_m_*) may vary among species (Fig. SB-1).^1,2,4,64^  However, the differences in mineralization rates among species are not well established and, to our knowledge, mineralization rate studies have not been performed in horses. Consequently, we cannot directly estimate *k_m_* for our horses from equine data. Limited evidence suggests exercise increases the mineralization rate, however this dependence is also not well described.^66^ Therefore, we will assume a constant *k_m_* that is unaffected by exercise. The value we select (k_m_ = 0.00505 days^-1;^ Table 1) is the average *k_m_* from cortical and cancellous data from ewes estimated via root-mean-square error curve-fitting to normalized data from Bala, et. al, 2010 to Eqn. 3. We also assume that the same mineralization law (equation (3)) holds in both the damaged and undamaged volume compartments. There is evidence that damaged tissue can reach a higher peak mineral density than undamaged tissue;^67^ however, the relationship between mineral peaks in damaged and undamaged tissue is not well established. Therefore, we assume that k_m_ is unaffected by the presence (or absence) of tissue damage.

***Probability of Resorption***

We assume that resorption of mineralized bone is independent of the amount of time spent in its compartment. Physically, this models resorption as independent of the mineral content accumulated during secondary mineralization (i.e., the only requirement for resorption is for TMD > *TMD_o_*). An exponential probability distribution (Eqn. (S2-1)) is the unique solution for a probability distribution with these properties as it is a memoryless distribution.^68^ The average time to an event governed by an exponential probability distribution is 1/*λ*.^69^ Therefore, in equation (S2-1), 1/*λ* is the average time to resorption. This is convenient because the average residence time in a compartment is reciprocal of the sum of outflow rates (1/Σ*k_i,out_)*.^34,70^ Therefore, we identify λ = Σk_i,out_. For undamaged mineralized bone, λ = k_1_ + k_4_ (which represents tissue leaving the compartment due to resorption (k_1_) or by being damaged (k_4_)) and for damaged mineralized bone λ = k_5_. Notice if k_5_ > k_1_, resorption of BV_D_/*TV* occurs (on average) faster than BV_UD_/*TV* bone. This means that k_5_ > k_1_ is targeted remodeling*,* which is important to note because targeted remodeling of damaged tissue is well established in bone turnover.^9,43^

$P\left( t \right)=\lambda e^{-\lambda t}$ (S2-1)

***Average Mineralization***

The average TMD within a mineralized compartment can now be defined using equation (2), using m(t) and P(t) defined by equations (3) and (S2-1). Equation (S2-2) defines the average TMD within the undamaged mineralized bone volume fraction (BV_UD_/*TV*) and equation (S2-3) describes the average TMD within the damaged mineralized bone volume fraction (BV_D_/*TV*). In equation (S2-3), the m(t) profile starts at t = 1/k_4_ rather than at t = 0; 1/k_4_ is the average time tissue spends in the undamaged compartment before transferring to the damaged compartment. This time shift sets the starting TMD of tissue in the damaged compartment to the average TMD accumulated after 1/k_4_ days in the undamaged compartment.

$TMD_{BV_{UD}}= \int_{0}^{\infty} m\left( t \right)\left( k_{1}+k_{4} \right)e^{-\left( k_{1}+k_{4} \right)t}dt=\frac{k_{1}+k_{4}}{k_{1}+{k_{4}+ k}_{m}}TMD_{o}+ \frac{k_{m}}{k_{1}{{+ k}_{4}+ k}_{m}}TMD_{max}$ (S2-2)

$TMD_{BV_{D}} = \int_{0}^{\infty} m\left( t+\frac{1}{k_{4}} \right)k_{5}e^{-k_{5}t}dt =TMD_{max} + \frac{k_{5}e^{-\frac{k_{m}}{k_{4}}}(TMD_{o}-TMD_{max})}{k_{5}+k_{m}}$ (S2-3)

**Supplementary Information 3: Selection of a Fixed Primary Mineralization Rate (k_3_)**

In the proposed compartment model, mineralized bone tissue (BV_D_ or BV_UD_) was assumed to have completed primary mineralization. This assumption requires two processes to occur before tissue transfers out of the osteoid volume (*OV*) compartment into the mineralized bone compartment (BV_UD_): osteoid maturation and primary mineralization. Osteoid maturation time depends on the time needed to complete the aspects of osteoid maturation (e.g., collagen crosslinking), which can depend upon animal health. For example, Vitamin D-deficiencies slow the osteoid maturation process.^71^ Primary mineralization is assumed to occur within hours or days;^3^ however, the exact time to complete the process is unknown.

Histologically, two terms are commonly used to define the time that osteoid remains un-mineralized: mineralization lag time (MLT) and osteoid maturation time (OMT). By definition OMT < MLT, but assuming that all horses in our population are healthy and that matrix maturation is the main determinant for the onset of mineralization, OMT = MLT.^3,72^ The effects of exercise on OMT or MLT are not described in the literature. Additionally, exercise does not appear to affect the overall primary mineralization rate.^66^ Therefore, we will assume that both osteoid maturation and primary mineralization are unaffected by mechanical loading (exercise) and that k_3_ was the same for all study horses.

Osteoid mineralization rate is not defined on standard lists of bone histology variables.^73^ However, Parfitt defined osteoid mineralization rate as the inverse of MLT, so we could assume that the average time required to complete primary mineralization (1/k_3_) is included in MLT measurements (i.e., k_3_ = 1/MLT).^74^ To our knowledge, there are no estimates for either OMT or MLT for horses and it is unknown if this rate varies among species. However, previous work reports MLT of 26-58 days in women,^74^ 9.5-10.7 days in female dogs,^75^ and 1.2-1.4 days in rat.^64,71^ In context of the compartment model, using k_3_ = 1/MLT with these values indicates the average residence time in the osteoid volume compartment is between 1.2-58 days and k_3_ is between 0.03-0.83 days^-1^. Since we do not have a direct estimate for MLT, and therefore k_3_, in horses we must assume the value for k_3_ to solve for model rate constants. We will determine the minimum feasible k_3_ that allows acceptable solutions (i.e., non-negative rate constants) for each Non-Damaged ROI (this need not be done in the Damaged ROI because of the solving method, outlined below). We also will test whether different assumptions of k_3_ change the significant correlations between the rate constants and measurements of exercise intensity.

***Effect of k_3_ on Steady-State Solutions***

Group did not have a significant effect on the smallest feasible k_3_ observed in the Non-Damaged ROI. In the Non-Damaged ROI, the least squares mean and standard error of the smallest feasible *k_3_* were 0.11 ± 0.07 days^-1^, 0.24 ± 0.07 days^-1^, and 0.33 ± 0.07 days^-1^ for the Control, Case CLI, and Case FX groups. There was a significant pairwise difference in the smallest feasible *k_3_* between the FXs and CTRLs in the Non-Damaged ROI. The smallest feasible *k_3_* that allowed for *k_2_* > 0 in all samples was 1.024 days^-1^.

The choice of k_3_ = 1.024, 10, or 100 days^-1^ did not affect the significant correlations between *k_1_* and k_2_ with BVF and TMD, exercise relationships to k_1_ (which was expected because k_1_ did not depend on k_3_), or exercise relationships with *k_2_* in the Non-Damaged ROI. However, the regressions performed with *k_3_* = 100 days^-1^ tended to have higher r^2^ compared to those performed with k_3_ = 1.024 or 10 days^-1^.

In the Damaged ROI, using *k_3_* = 1.024 days^-1^ resulted in higher root-mean-square error for the predicted volume fractions compared to *k_3_* of 10 or 100 days^-1^. Additionally, k_3_ = 1.024 or 10 days^-1^ resulted in one sample with *k_5_* < k_1_; this was undesirable as we require *k_5_* > k_1_ for targeted remodeling. With *k_3_* = 100 days^-1^, *k_5_* > *k_1_* for all samples. The choice of *k_3_* did not affect the correlations among *k_1_*, *k_2_*_,_ and BVF, *BV_D_*/*TV*, *BV_UD_*/*TV* and TMD in the Damaged ROI. However, setting *k_3_* = 1.024 days^-1^ gave significant correlations between k_5_ and BVF and TMD that were not present with *k_3_* = 10 or 100 and removed a significant correlation between k_4_ and Cr.Af that was observed with *k_3_* = 10 and 100. Because *k_3_* = 100 allowed for targeted remodeling in the Damaged ROI, and k_3_ had a limited effect on correlations to morphologic variables in both ROIs, all remaining results use *k_3_* = 100 days^-1^.

**Supplementary Information 4: ANOVA & Exercise History Regressions**

**Table S4-1** Least Square Means (Standard Errors) from linear mixed model or Raw Means (Standard Deviation) of a ranked linear mixed model^¶^ for tissue properties. Significant effects are indicated by variable superscripts: ^†^(ROI), ^§^(Group), ^‡^(interaction of Group and ROI). Simple comparisons (F-Test) of ROIs within a Group (down Column) are indicated by superscripts (α,β). Simple comparisons of Groups within an ROI (across Row) are indicated by subscripts (A,B,C). For all comparisons, significant simple comparisons are indicated with a * and variables that do not share a subscript or superscript are significantly different at p < 0.05.

|  | **BVF (%)** ^‡^ | | | |
| --- | --- | --- | --- | --- |
|  | **FX^*^** | **CLI** | | **CTRL^*^** |
| Damaged^*^ | 90 (2)^A^_α_ | 96 (2)^A,B^_α_ | | 98(2)^B^_α_ |
| Non-Damaged^*^ | 96 (2)^A^_β_ | 97 (2)^A^_α_ | | 90 (2)^A^_β_ |
|  | **TMD (mgHA/ccm)** ^†, §^ | | | |
|  | **FX^*^** | **CLI^*^** | **CTRL^*^** | |
| Damaged^*^ | 790.55 (5.8) ^A^_α_ | 809.15 (5.8)  ^B^_α_ | 812.37 (5.8)  ^B^_α_ | |
| Non-Damaged^*^ | 842.63 (5.8) ^A^_β_ | 852.94 (5.8) ^A^_β_ | 843.47 (5.8) ^A^_β_ | |
|  | **Cr.Af (mm^2^/mm^2^)** ^¶, †, §, ‡^ | | | |
|  | **FX^*^** | **CLI^*^** | **CTRL^*^** | |
| Damaged^*^ | .020 (0.017) ^A^_α_ | .008 (0.006) ^B^_α_ | 0.004 (0.004) ^B^_α_ | |
| Non-Damaged | 0 (0) ^A^_β_ | 0 (0) ^A^_β_ | 0 (0) ^A^_β_ | |

**Table S4-2** Spearman correlation coefficients (p-value) among calculated steady-state rate constants and morphologic data. Correlations were calculated separately in the Non-Damaged and Damaged regions of interest (ROI). Significant correlations are shown in bold (p < 0.05). Correlations that are were not calculated are indicated with “N/A”.

|  | **Non-Damaged ROI** | | **Damaged**  **ROI** | | | |
| --- | --- | --- | --- | --- | --- | --- |
|  | *k_1_*  (days^-1^) | *k_2_*  (days^-1^) | *k_1_*  (days^-1^) | *k_2_*  (days^-1^) | *k_4_*  (days^-1^) | *k_5_*  (days^-1^) |
| Bone Volume Fraction | **-0.42 (0.02)** | **0.98** (<0.0001) | **-0.74** (<0.0001) | **1** (<0.0001) | -0.08 (0.68) | **0.38** (0.05) |
| Tissue Mineral Density  (mgHA/ccm) | **-1** (<0.0001) | 0.29 (0.12) | **-0.84** (<0.0001) | **0.81** (<0.0001) | -0.08 (0.68) | **0.37** (0.05) |
| Stained Linear Microcrack Area Fraction  (mm^2^/mm^2^) | N/A | N/A | 0.06 (0.76) | -0.35 (0.07) | **0.82** (<0.0001) | 0.11 (0.56) |
| *k_1_*  (days^-1^) | 1 | -0.29 (0.12) | N/A | **-0.74** (<0.0001) | -0.31 (0.11) | **-0.64** (0.0003) |
| *k_2_*  (days^-1^) | -0.29 (0.12) | N/A | **-0.74** (<0.0001) | N/A | -0.07 (0.73) | **0.39** (0.04) |
| *k_4_*  (days^-1^) | N/A | N/A | -0.31 (0.11) | -0.07 (0.73) | N/A | **0.54** (0.003) |

**Table S4-3** Summary [Raw Mean (Standard Deviation)] of derived steady-state rate constants. From a linear mixed model on ranked data, significant effects are indicated by variable superscripts: ^†^(ROI), ^§^(Group), ^‡^(interaction of ROI and Group). For each steady-state rate constant, values that share a superscript are not statistically different at p < 0.05. These calculations were performed with k_3_ = 100 days^-1^ and other constants as defined in Table 1. Note that day^-1^ indicates the rate constants are measured on a “per day” basis, i.e. 0.006 days^-1^ is 0.006 per day.

|  |  | **Control**  **(CTRL)** | **Case**  **Contralateral Intact**  **(CLI)** | **Case**  **Fractured**  **(FX)** |
| --- | --- | --- | --- | --- |
| ***k_1_***^†^  (days^-1^) | Damaged  ROI | 0.0063 (0.0005)^A,B^ | 0.0058 (0.0021) ^A,B^ | 0.0068 (0.0017)^A^ |
|  | Non-Damaged  ROI | 0.0053 (0.0005)^B,C^ | 0.0051 (0.0006)^C^ | 0.0054 (0.0008) ^B,C^ |
| ***k_2_***^‡^  (days^-1^) | Damaged  ROI | 0.8872 (0.722)^A^ | 0.4534 (0.5563)^A,B^ | 0.4816 (1.1887)^B^ |
|  | Non-Damaged  ROI | 0.1150 (0.120)^B^ | 0.2380 (0.1508)^A,B^ | 0.3364 (0.3222)^A,B^ |
| ***k_4_***^†^  (days^-1^) | Damaged  ROI | 0.0002 (0.0003) ^A^ | 0.0010 (0.0018)^A^ | 0.0011 (0.0015)^A^ |
|  | Non-Damaged  ROI | 0 (0)^B^ | 0 (0)^B^ | 0 (0)^B^ |
| ***k_5_***^†^  (days^-1^) | Damaged  ROI | 0.1658 (0.2460)^A^ | 0.2222 (0.5747)^A^ | 0.0422 (0.0213)^A^ |
|  | Non-Damaged  ROI | 0 (0)^B^ | 0 (0)^B^ | 0 (0)^B^ |

**Table S4-4**: Robust linear regressions results [Slope (R^2^)] between k_1_ or ln(k_1_) and exercise variables.^25^ Regressions were performed separately in the Damaged and Non-Damaged regions of interest (ROI). All reported results were significant at p < 0.05. Layup is defined as 60 or more days when a horse had no official events and active periods exclude times when a horse was in layup. Furlongs are 1/8 mile (220 yards) and are a standard measurement unit used in horse racing.

| **Exercise Variable** | | **Damaged ROI** | | **Non-Damaged ROI** | |
| --- | --- | --- | --- | --- | --- |
| **Type** | **Variable (Units)** | ***k_1_*** | **ln(*k_1_*)** | ***k_1_*** | **ln(*k_1_*)** |
| Before Death | Races 10 Months Before Death (races) | . | . | -0.0001 (0.11) | . |
| Exercise Intensity | Days Between Events During Active Training  (days) | . | . | -0.0001 (0.14) | . |
|  | Days Between Works During Active Training  (days) | . | . | -0.0001 (0.31) | . |
|  | Events per Year during Active Training  (events/yr) | . | . | 0.000036 (0.16) | . |
|  | Furlongs Worked per Month of Active Training  (furlong/mo) | . | . | 0.0001 (0.23) | 0.0065 (0.17) |
|  | Works Per Year of Active Training  (works/yr) | . | . | 0.000029 (0.18) | 0.0019 (0.14) |
| Layup | Percent Career in Layup  (%) | . | . | 0.000009 (0.17) | 0.0007 (0.15) |

**Table S4-5:** Robust linear regressions results [Slope (R^2^)] between k_2_ or ln(*k_2_*) and exercise variables.^25^ Regressions were performed in both the Damaged and Non-Damaged regions of interest (ROI). All reported results were significant at p < 0.05. Events include all official timed workouts (works) and races, a layup is defined as 60 or more days when a horse had no official events, and active periods exclude times when a horse was in layup. Furlongs are 1/8 mile (220 yards) and are a standard measurement unit used in horse racing.

| **Exercise Variables** | | **Damaged ROI** | | **Non-Damaged ROI** | |
| --- | --- | --- | --- | --- | --- |
| **Type** | **Variable Name** | ***k_2_*** | **ln(*k_2_*)** | ***k_2_*** | **ln(*k_2_*)** |
| Before Death | Days between death and previous event (days) | . | . | . | -0.0049 (0.32) |
|  | Races 1 Month Before Death (races) | . | . | 0.0684 (0.17) |  |
|  | Races 6 Month Before Death (races) | . | . | 0.022 (0.23) | 0.0607 (0.19) |
|  | Races 8 Month Before Death(races) | . | . | 0.0189 (0.21) | . |
|  | Races 10 Month Before Death(races) | . | . | 0.0152 (0.17) | . |
|  | Events 1 Month Before Death (events) | -0.216 (0.23) | -0.482 (0.32) | . | . |
|  | Events 2 Months Before Death (events) | -0.131 (0.3) | -0.29 (0.41) | . | . |
|  | Events 4 Months Before Death (events) | -0.06 (0.28) | -0.134 (0.38) | . | . |
|  | Events 6 Months Before Death (events) | -0.042 (0.2) | -0.093 (0.31) | . | . |
|  | Events 8 Months Before Death (events) | -0.031 (0.15) | -0.074 (0.28) | . | . |
|  | Events 10 Months Before Death (events) | -0.026 (0.15) | -0.068 (0.31) | . | . |
|  | Events 1 Year Before Death (events) | -0.022 (0.11) | -0.067 (0.29) | . | . |
|  | Furlongs 1 Month Before Death (furlongs) | -0.035 (0.17) | -0.07 (0.22) | . | . |
|  | Furlongs 2 Months Before Death (furlongs) | -0.021 (0.22) | -0.046 (0.31) | . | . |
|  | Furlongs 4 Months Before Death (furlongs) | -0.009 (0.17) | -0.021 (0.25) | . | . |
|  | Furlongs 10 Months Before Death (furlongs) | . | -0.01 (0.22) | . | . |
|  | Furlongs 1 Year Before Death (furlongs) | . | -0.009 (0.19) | . | . |
|  | Works 2 Months Before Death (works) | . | -0.207 (0.21) | . | . |
|  | Works 4 Months Before Death (works) | -0.061 (0.2) | -0.165 (0.37) | . | . |
|  | Works 6 Months Before Death (works) | -0.049 (0.16) | . | . | . |
|  | Works 8 Months Before Death (works) | . | -0.116 (0.32) | . | . |
|  | Works 10 Months Before Death (furlongs) | . | -0.094 (0.32) | . | . |
|  | Works 1 Year Before Death (furlongs) | . | -0.083 (0.26) | . | . |
| Exercise Intensity | Days Between Events (days) | . | 0.069 (0.26) | -0.0071 (0.19) | -0.0356 (0.38) |
|  | Days Between Works During Active Training (days) | **.** | . | 0.0108 (0.26) |  |
|  | Days Between Works (days) | 0.035 (0.15) | 0.073 (0.29) |  |  |
|  | Events per Year during Active Training (events/yr) |  |  |  |  |
|  | Events per Year (events/yr) | -0.027 (0.22) | -0.051 (0.26) | 0.0047 (0.17) | 0.0189 (0.21) |
|  | Furlong per Month (furlong/mo) | -0.07 (0.19) | -0.125 (0.21) | 0.0149 (0.20) | 0.058 (0.25) |
|  | Furlongs Raced per Month (furlong/mo) |  |  | 0.0239 (0.19) |  |
|  | Furlongs Worked per Month (furlong/mo) | . | -0.182 (0.24) |  |  |
|  | Races per year (races/yr) |  |  | 0.0129 (0.19) |  |
|  | Works per year (works/yr) | . | -0.049 (0.19) |  |  |
| Layup | Days since last layup (days) | -0.002 (0.12) | -0.005 (0.3) |  |  |
|  | Events Since Last Layup (events) | -0.018 (0.15) | -0.055 (0.32) |  |  |
|  | Number of Layups (layup) | . | . | -0.0498 (0.21) | -0.2095 (0.32) |
|  | Total Time in Layup (days) | . | 0.003 (0.19) |  |  |
|  | Average Layup Length (days) | . | 0.006 (0.18) | -0.0006 (0.17) |  |
|  | Percent Career in Layup (%) | 0.012 (0.23) | 0.022 (0.22) | -0.0028 (0.27) | -0.0128 (0.45) |
| Lifetime | Age (years) | . | 0.749 (0.04) |  |  |

**Table S4-6:** Robust linear regressions results [Slope (R^2^)] between *k_4_* and ln(k_4_) and exercise variables.^25^ All reported results were significant at p < 0.05. Note that: Events include all official timed workouts (works) and races. Events include all official timed workouts (works) and races, a layup is defined as 60 or more days when a horse had no official events, and active periods exclude times when a horse was in layup. Furlongs are 1/8 mile (220 yards) and are a standard measurement unit used in horse racing.

| **Exercise Variables** | | **Damaged ROI** | |
| --- | --- | --- | --- |
| **Type** | **Variable Name** | ***k_4_*** | **ln(*k_4_*)** |
| Layup | Number of Works (works) | 0.000012 (0) | . |
|  | Average Layup Length (days) | . | 0.008 (0.13) |
| Exercise  Intensity | Days Between Races During Active Training (days) | . | 0.088 (0.19) |
| Exercise Before  Death | Works 4 Months Before Death (works) | 0.000036 (0.20) | . |

**Supplementary Information 5: Sensitivity of k_1_ to Input Distributions in Non-Damaged ROI**

Borgonovo sensitivity analysis indicated that the output distribution of k_1_ in the Non-Damaged ROI (from equation (8)) was most sensitive to *k_m_*, followed by *TMD_ROI_*, and then *TMD_max_*. For these calculations, *TMDo* was fixed to 540 mgHA/ccm because we did not have a distribution for the value. *TMD_ROI_* was modeled as a Gaussian distribution with mean and standard deviation of the TMD measured in the Non-Damaged Region in all PSBs: 846.34 ± 20.14 mgHA/ccm. TMD_max_ was modeled as a Gaussian distribution with mean and standard deviation of the maximum TMD measured in all PSBs: 1163.70 ± 21.27 mgHA/ccm. Mineralization rate (*k_m_*) was modeled as a Gaussian distribution using curve-fits of equation (3) to data from cortical and cancellous sheep bone with a mean and standard deviation of 0.00505 ± 0.0009 days^-1^.^1^ Mineralization rate was also modeled as a Gaussian or uniform random distribution using curve-fits of equation (3) to data from young rats,^64^ rabbits,^2^ and sheep^1^ (mean: 0.31 days^-1^, standard deviation: 0.61 days^-1^). Regardless of the *k_m_* distribution, the output distribution of *k_1_* was most affected by the distribution of *k_m_*, and less affected by the distributions of *TMD_ROI_* and *TMD_max_*.

**Supplementary References**

References not listed in the main manuscript are found below:

1. Ruffoni, D., Fratzl, P., Roschger, P., Klaushofer, K. & Weinkamer, R. The bone mineralization
    density distribution as a fingerprint of the mineralization process. *Bone* **40**, 1308–1319 (2007).
2. Lukas, C. *et al.* Mineralization kinetics in murine trabecular bone quantified by time-lapsed in vivo
    micro-computed tomography. *Bone* **56**, 55–60 (2013).
3. Aruwajoye, O. O. *et al.* Microcrack density and nanomechanical properties in the subchondral region
    of the immature piglet femoral head following ischemic osteonecrosis. *Bone* **52**, 632–639
    (2013).
4. Leemis, L. M. & Mcqueston, J. T. Univariate Distribution Relationships. *Am. Stat.* **62**, 45–53 (2008).
5. Jowett, G. H. The Exponential Distribution and Its Applications. *Inc. Stat.* **8**, 89 (1958).
6. Matis, J. H. & Wehrly, T. E. On the Use of Residence Time Moments in the Statistical Analysis of Age-
    Dependent Stochastic Compartmental Systems. in *Mathematics in Biology and Medicine.*
    *Lecture Notes in Biomathematics, vol 57.* (eds. Capasso, Grosso & Paveri-Fontana) 386–398
    (Springer, Berlin, Heidelberg, 1985). doi:10.1007/978-3-642-93287-8_53
7. Baylink, D., Stauffer, M., Wergedal, J. & Rich, C. Formation, mineralization, and resorption of bone in
    vitamin D—deficient rats. *J. Clin. Invest.* **49**, 1122–1134 (1970).
8. Parfitt, M. A. *et al.* Bone Histomorphometry : Standardization of Nomenclature, Symbols, and Units.
    *J. Bone Miner. Res.* **2**, 595–610 (1987).
9. Dempster, D. W. *et al.* Standardized nomenclature, symbols, and units for bone histomorphometry:
    A 2012 update of the report of the ASBMR Histomorphometry Nomenclature Committee. *J.* *Bone Miner. Res.* **28**, 2–17 (2013).
10. Parfitt, A. M., Han, Z.-H., Palnitkar, S., Rao, D. S. & Nelson, D. Effects of Ethnicity and Age or
     Menopause on the Remodeling and Turnover of Iliac Bone: Implications for Mechanisms of
     Bone Loss. *J. Bone Miner. Res.* **12**, 498–508 (1997).
11. High, W. B., Capen, C. C. & Black, H. E. The effects of 1,25-dihydroxycholecalciferol, parathyroid
     hormone, and thyroxine on trabecular bone remodeling in adult dogs. A histomorphometric
     study. *Am. J. Pathol.* **105**, 279–87 (1981).
